# Toward a Constructivist Model of Radicalization and Deradicalization: A Conceptual and Methodological Proposal

**DOI:** 10.3389/fpsyg.2019.00412

**Published:** 2019-03-06

**Authors:** David A. Winter, Guillem Feixas

**Affiliations:** ^1^ University of Hertfordshire, Hatfield, United Kingdom; ^2^ Department of Clinical Psychology and Psychobiology, Institute of Neurosciences, University of Barcelona, Barcelona, Spain

**Keywords:** repertory grid, personal construct, identity, sense of identity, identity fusion, uncertainty, construal

## Abstract

This article identifies common features of existing models of radicalization and deradicalization, such as the transition from uncertainty to certainty, before integrating these in a model based upon personal construct theory. It is proposed that the personal construct concepts of validation and invalidation are particularly relevant to processes of identity change such as radicalization and deradicalization. Thus, it is argued that radicalization occurs when major invalidation of an individual’s construing is followed by the development of a new radicalized view of the world that provides a turning point in his or her sense of identity and a more structured and certain view of the world. There is likely to be seeking out of validation for this view in interactions with others who share similar views or by extorting evidence for the individual’s radical constructions. These constructions are likely to involve extreme negative views of another group, by contrast to members of which, and possibly by taking extreme action against this group, the individual’s new self-construction may become further defined. These same processes can be seen to operate in deradicalization, and it will therefore be argued that the model has implications for the development of deradicalization programs. A further advantage of the model is that it has an associated personal construct methodology, particularly repertory grid technique, that may be used to investigate processes of radicalization and deradicalization. As illustrations of such investigations, results will be summarized from a repertory grid study of Salafist Muslims in Tunisia, some of whom had returned from fighting in Syria, and an analysis of the writings of the Norwegian mass murderer Anders Breivik. The findings of these investigations are argued to be consistent with the personal construct model of radicalization and deradicalization.

## Introduction

The crucial importance of increasing our understanding of radicalization is highlighted by definitions of the term in strategies and policy recommendations concerning the countering of terrorism and violent extremism. For example, the UK government has defined it as “The process by which people come to support terrorism and violent extremism and, in some cases, then join terrorist groups” (HM Government, 2009, p. 11), while a European Union definition is “a phenomenon of people who regard the use of violence as legitimate and/or use violence themselves in order to achieve their political objectives which undermine the democratic legal order and the fundamental rights on which it is based” (European Union Committee of the Regions, 2016, p. 4). These definitions reflect concerns about young people whose radicalization has involved choosing the path of what has been somewhat inaccurately described (Post et al., 2009) as violent Jihad, or of violence with very different ideological roots. However, people may develop radical views without supporting, or participating in, violence, and indeed in certain areas of our lives most of us hold views which might be considered extreme, at least by some others (Neumann, 2003; Mandel, 2010; Borum, 2011a; Bartlett and Miller, 2012). Also, what is radical in one social, cultural, or temporal context may not be so regarded in another.

This paper will concern itself with the development by an individual of all-pervading views which may be considered radical in his or her social context, and with the circumstances in which these may lead to violent action, as well as with the converse process of deradicalization. Previous research has sought in vain for a profile, in terms of psychopathology, personality features, or economic deprivation, of people who have become radicalized and/or engaged in terrorism (Horgan, 2003; McCauley, 2004; Silke, 2008; Gartenstein-Ross and Grossman, 2009; McGilloway et al., 2015). Nevertheless, various explanations of the process of radicalization have been proposed, and we shall consider their common features before outlining an alternative model.

It has been suggested that prior to radicalization the individual experiences a state of uncertainty about the self and the world (Hogg et al., 2010, 2013; Hogg, 2012; Doosje et al., 2013; Klein and Kruglanski, 2013; Meeus, 2015), and existential anxiety (McBride, 2011). Among the contributing factors to this may be perceived relative deprivation, for example for immigrants in comparison to their status in their countries of origin or to indigenous people of the host country, or failure to fulfill one’s aspirations (Moghaddam, 2005); experienced prejudice and perceived exclusion from an ingroup (Stroink, 2007; Victoroff et al., 2012); alienation (Horgan, 2008; Wilner and Dubouloz, 2010); threats to one’s collective identity as a result of globalization (Moghaddam, 2012); and mortality salience (Pyszczynski et al., 2006). These factors may include conditions in the individual’s social context or personal crises or “disorienting dilemmas” that act as “transformative triggers” (Wilner and Dubouloz, 2010) and “turning points” (McAdams and Bowman, 2001) or provide “cognitive openings” (Wiktorowicz, 2005) and a “readiness to change.” In such situations, the person may be particularly prone to identity transformation. As indicated by social identity theory (Tajfel and Jones, 1979), this, coupled with an increase in self-esteem, may be facilitated by identification with an available social category, such as might be provided by a radical group (Taylor and Louis, 2004; Dalgaard-Nielsen, 2010). The individual may become increasingly socialized into this group (Silke, 2008; Dalgaard-Nielsen, 2010) by face-to-face contact or internet “echo rooms” (Geeraerts, 2012), while becoming relatively isolated from wider society, including his or her previous social network. The radical group, with its “high entativity” (coherence) and extreme, clear, and simple view of the world (Savage and Liht, 2008; Liht and Savage, 2013), perhaps expressed in terms of a “sacred canopy” of religious beliefs (Berger and Luckmann, 1967; Griffin, 2012), may provide a sense of certainty (Hogg, 2012) about the world, the future, and the self (and even about life after death in some cases!).

The individual’s identity may then become “fused” with that of the group, increasing his or her willingness to die for the group if it is threatened (Swann et al., 2009, 2014; Whitehouse et al., 2014; Atran, 2016), especially if it has a “culture of martyrdom” (Gill, 2007). This may be particularly so if the worldview embraced involves grievances toward a demonized, hated, and dehumanized other group (Bandura, 1999; McCauley and Moskalenko, 2011; Monahan, 2012) because one’s identity may be strengthened by contrast with the other (Herriott, 2009). As Alford (2005) puts it, “Hatred is self-structure on the cheap” (p. 252) and “hate gives meaning to life” (p. 239). Involvement in violent extremism may also fulfill the person’s “quest for significance” (Kruglanski et al., 2009, 2014; Webber et al., 2017) and status (Bartlett and Miller, 2012) in his or her particular social context. Borum (2003, 2011b) considers that violent action against a vilified group is often the end of a process in which this group is blamed for socioeconomic deprivation experienced by the group with which a terrorist identifies and which is perceived as unjust.

In short, previous analyses of radicalization suggest that a state of uncertainty may make an individual open to identification with a group whose radical views, including demonization of another group, provide a new sense of certainty, possibly including a justification for violent action against the other group.

## Personal Construct Theory

In his writings on radicalization and violent extremism, Borum (2003), p. 8, stresses understanding of the terrorist’s “mind-set” and “own internal ‘map’ of reality” to anticipate his or her actions. The model which we shall propose aims to understand the radicalized person’s “own internal ‘map’ of reality” by using the concepts of Kelly (1955) personal construct theory (PCT). One of the first examples of a constructivist approach in psychology, this asserts that people are primarily concerned with anticipating, and giving meaning to, their worlds, which they do by developing hierarchically organized systems of bipolar personal constructs (e.g., “good” versus “evil”) in which some constructs are more superordinate, or important, than others. Not only individuals, but also families, groups, societies, and cultures, may be viewed as operating with their own construct systems, and the theory is thus as applicable to social as to individual constructions and processes (Procter, 2016).

Their constructs allow people to discriminate between events, and essentially provide the goggles through which the world is viewed and the basis for the predictions and choices that people make. The individual’s constructions of aspects of the world may be subsequently validated or invalidated. The optimally functioning person will generally revise his or her constructions if they are invalidated, and construing is therefore a cyclical process [referred to by Kelly (1970) as the Experience Cycle] in which the individual, similar to a scientist, constantly formulates hypotheses about his or her world, tests them out, refines them if necessary, and retests them. Kelly (1955) also outlined two other cyclical processes central to the person’s construction and reconstruction of their world. In one of these, the Creativity Cycle, the person’s construing becomes looser, or more vague and flexible, allowing constructs to be “reshuffled,” before tightening again, enabling new predictions to be made and tested. The other cycle, the Circumspection-Preemption-Control Cycle, concerns decision-making and involves the person considering all the constructs relevant to a decision, focusing on the most superordinate of these, and selecting one pole of this construct as the basis of his or her decision.

Kelly (1969a) viewed the individual’s construct system as providing his or her identity, and indicated that certain superordinate constructs, referred to as core constructs, are particularly central in this regard. The prospect of a comprehensive challenge to a person’s core constructs will provoke threat, while guilt will be experienced if the person experiences behaving in a way that is discrepant from his or her “core role,” or customary view of the self. If the person finds that his or her constructs do not enable the anticipation of events, he or she will experience anxiety. While, as we have seen, the person may be able to reduce anxiety by reconstruing events if his or her constructions are invalidated, some types of construct system are more conducive to the accommodation of reconstruing than are others. If, for example, a person’s view of the world is unidimensional and undifferentiated, no alternative constructions of events may be possible and the impact of invalidation may be massive in that it may lead to structural collapse of the whole system. Sometimes, therefore, rather than changing his or her constructions after invalidation, the person will attempt to change the world to make it fit with these constructions, a process that Kelly (1955) described as hostility. Other strategies for dealing with, or attempting to avoid, invalidation include constricting one’s world to exclude events that may be incompatible with one’s construing, or conversely dilating one’s world, throwing oneself into new experiences such that this may lead to development of ways of construing that will resolve the previous inconsistencies.

PCT views sociality, construing the other person’s construction processes and essentially attempting to see the world through his or her eyes, as the basis of interpersonal relationships. Similarly, Kelly (1955) enjoined the psychologist to take a “credulous attitude” to the other person, taking his or her view of the world seriously and at face value even if one disagrees with it. Viewed from the perspective of the individual’s construct system, even the most apparently self-destructive choices can be comprehended as attempts by the individual to predict the world or to avoid invalidation of construing and the accompanying anxiety and threat (Winter, 1992). For example, some people may take the choice of suicide because their lives are so chaotic that death provides the only certainty (Neimeyer and Winter, 2006). PCT may also provide an understanding of choices and actions that are destructive of others (although this, of course, does not imply condoning them), and its concepts have been used in formulations of cases of extreme violence, including homicide (Winter, 2007, 2016).

Kelly’s “first principle” was that “if you do not know what is wrong with a person, ask him; he may tell you” (Kelly, 1955, pp. 322–323). Spinzak has taken this advice in exploring violent extremism in that he reportedly remarked that “the best way to find out what leads people along the path of extremism, what leads people to be willing to kill in the name of their cause, is – to ask them!” (Post et al., 2003, p. 171). We shall now consider how the PCT concepts that we have described may be applied to the process of radicalization, including violent extremism, as it has been outlined in the previous literature that we have reviewed. We shall then describe PCT methods that may be used to ask the person what has led him or her along this path.

## A PCT Model Of Radicalization

**Figure 1:**
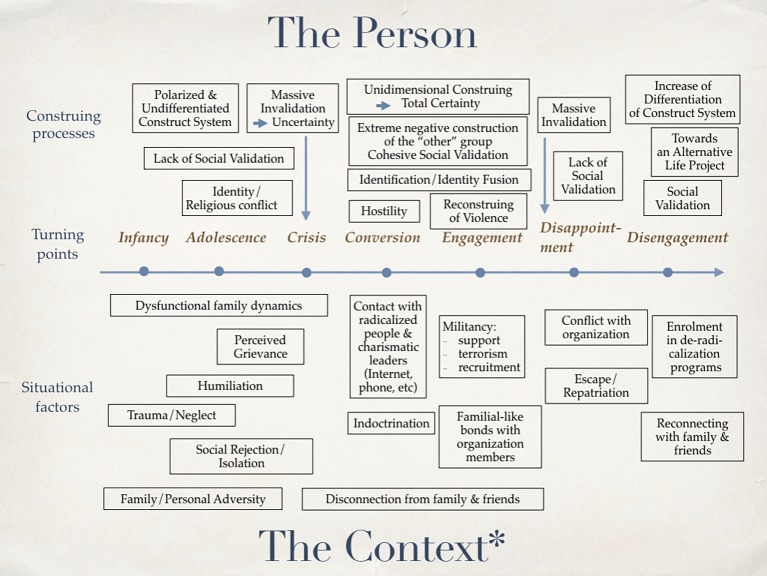
A personal construct heuristic model for the process of radicalization and deradicalization. Vertical arrows indicate “Transformative Trigger”. *Broader context includes social and political national/international conflicts, economic inequalities, religious fundamentalist confronted discourses (in worship places or web pages), social marginalization, and stigmatization.

From a PCT perspective, the pathway toward radicalization, which is presented in Figure 1, may be considered to consist of several stages.

1. *The radicalized individual has a history of invalidation of his/her construing, particularly in regard to core aspects of self-construing*.
  The various factors that in previous research have been identified as contributing to an individual’s state of uncertainty prior to radicalization may all be viewed as involving invalidation of the person’s construing. They may range from experiences in close personal relationships, for example at the level of family dynamics, to broader social adversities, such as rejection, prejudice, humiliation, and other perceived grievances. The individual’s resulting difficulty in construing his or her world will be experienced as anxiety, together with threat if core constructs are involved. There will also be guilt if the person’s experiences lead to dislodgement from his or her core role, for example by being denied the opportunities that would enable the self to continue to be viewed as successful or able to provide for his or her family.
2. This sometimes involves one or more episodes that lead to massive invalidation, and act as “transformative triggers.”
  Massive invalidation occurs when the superordinate structure of the construct system, including core constructs, become disconfirmed in a short period of time, leaving the system with no capacity to carry out its fundamental function of providing a structure with which to anticipate and understand events. From the PCT perspective, the resulting extreme uncertainty will be experienced as intense anxiety and as very threatening when core constructs are comprehensively challenged. The individual who is faced with these emotions, coupled perhaps with feelings of rage, desperation, sadness, and emptiness, will find life difficult to manage in both the short and long term.
3. The individual with a very undifferentiated (and thus inflexible) construct system may be particularly vulnerable to such invalidation and consequent structural collapse.
  An undifferentiated, or unidimensional, construct system offers a restricted view of events so that when a personal construction becomes invalidated the system does not provide alternative viewpoints which would permit a more complex understanding of events. The only possible reconstruction may be what Kelly (1969b) termed slot rattling, construing an event or person at the opposite pole of the construct dimension to that at which it was previously construed (e.g., “if John is not good then he must be bad,” no other alternatives are considered). If reconstruction is not possible the system collapses and fails in its main function: to provide an explanation of current events and, thus, a future perspective for upcoming events. The resulting extreme uncertainty may be experienced as chaos.
4. His/her radical beliefs, usually drawing upon available social constructions, allow the development of a “turning point” in his or her sense of identity with a more structured and certain view of the world.
  Faced with invalidation and uncertainty, the person may engage in cycles of construing and reconstruing in order to search for an alternative way of giving meaning to his or her experiences and reducing anxiety and threat. This is likely to be found in some socially available conceptual/ideological frame (or system of constructs) different from the one which has just been invalidated, such as a radical worldview. This view may then be held with 100% certainty, providing a firm new structure for, and means of anticipating, the individual’s world. Guilt will also be reduced if the person develops, and acts in accordance with, a new core role as a member of a radical group.
5. The development of an extreme negative construction of another group, which may be perceived as responsible for the individual’s invalidations, allows further definition of the self by contrast with this group.
  The individual’s new worldview is likely to involve an extreme negative construction of some other group. The nature of this group (which may, for example, be formed of infidels, homosexuals, immigrants or supporters of a particular football team) is in many ways irrelevant, but what is crucial is that it allows the person to structure an equally extreme positive identity by contrast to members of the vilified group. As stated by the personal construct theorist Neimeyer (2002), p. 298, italics in original, “*demonized others may be a critical condition for maintaining the phenomenological validity of our position*.”
6. The individual’s radical constructions are validated by contact with others who share similar views, often coupled with constriction of their previous social world to avoid further invalidation.
  Often, individuals in the process of radicalization will increasingly limit their social contacts to a selected group of individuals, to whom their identity is linked and who become the primary source of validation of their constructions. This may involve them actively seeking out contact with radical groups, but may also result from an active and systematic recruitment campaign by such a group. Becoming a “member” of that club comes with a number of social benefits and strong feelings of “oneness,” the phenomenon that Swann et al. (2014) described as “identity fusion”.
7. *The likelihood of acting upon radical beliefs, including violent actions, is greater in those individuals in whom beliefs in such actions provide the greatest increment in the structure of his/her view of the self.*
  In some radicalized individuals, taking extreme actions in accordance with their radical beliefs may enable their self-construing to become even more structured and consistent with their new core roles. This may, for example, involve actualizing an identity as a martyr for a religious cause or a warrior in search of ultimate justice. In all such cases, there is absolute certainty (even the certainty of death) and a definite course of action with no consideration of potential alternatives, no shades of grey.
8. Reconstruing of violence as acceptable may be necessary if the person is to engage in such acts without guilt (and indeed to experience guilt for not engaging in them).
  The person who commits an act of violence will be likely to experience guilt if the self has always been construed as gentle, but not if the violence can be construed as a legitimate aspect of the person’s self-construction, perhaps being seen as in pursuit of some worthy or “supreme” goal. Thus, for a person adopting a radical Jihadi identity violence might become not just legitimate but the privileged road to reach heaven. Not becoming committed to that path in one way or another might provoke guilt.
9. *His/her radical view of the world may be shored up by “hostility,” in* *Kelly* (1955) *sense of extorting evidence for the individual’s constructions.*
  Since reality is complex (and with some areas of uncertainty), the certainty provided by a radical construct system can only be maintained if the person ignores invalidation and/or manufactures validation. Thus, Kellyan hostility permits the disregarding of any evidence which might invalidate the newly acquired radical identity and creating instances which conform to these constructions of self and others.
10. Similar processes may operate in members of the “other” group, creating a vicious cycle of extreme construing based on mutual validation of extreme negative views of the other.
  Hostility may become a cyclical process in which two groups each act in such a way that provokes behavior that validates each group’s negative constructions of the other. The resulting conflict escalation may be a fundamental terrorist goal (Louis and Taylor, 2002), a strategy that has been referred to as “jujitsu politics,” “using the enemy’s strength against him” (McCauley and Moskalenko, 2008, p. 427).
  The radicalization process need not involve an individual progressing through all the stages outlined above, and certain stages may be more relevant to some people than to others. For example, it should be noted that the individual who is socialized from childhood into a world view that involves extreme constructions of another group cannot really be considered to become radicalized by adopting such constructions. Nevertheless, the later stages of the proposed model can be used to explain why some such individuals proceed to a path of radical action which provides more structure and certainty in their self-construing than, for example, a non-violent self-construction. Similarly, these stages may also be relevant to radical actions taken by individuals who join a radical group because of reasons more associated, for example, with criminality or thrill seeking than with the adoption of radical beliefs, but for whom radical acts can then enhance their identities.
  Aspects of the proposed model can also be used to explain deradicalization. For example, an individual may become amenable to deradicalization after experiencing major invalidation in relation to his or her radical views. However, he or she is only likely to become deradicalized if a view of the world is available that provides at least as much structure and certainty as did the radical beliefs, and that is validated by others who share similar views. After the worldview full of certainty that he or she had embraced becomes invalidated all will appear to be confusing and the previously existing social validation will vanish. Assistance needed at this point will involve much support for developing a more complex, differentiated view of reality, social validation, and also engagement in creating a new sense of identity associated with a commitment to a life project which might be less deterministic and more prosocial.

## Personal Construct Methodology for the Exploration of Radicalization

A major advantage of PCT is that it provides not only theoretical concepts which encompass a wide variety of theoretical formulations about violent radicalization but also a very flexible methodology. The most widely used such method is the repertory grid (Fransella et al., 2004). The grid allows exploration of both the content and structure of a person’s construct system, including areas that, being at a low level of awareness, would be difficult to access by a conventional interview. Since it considers the individual’s own constructs rather than using pre-established items, it is well suited to investigations in different cultural settings, and it has been used in exploring the construing of survivors of a brutal civil war in Africa, including both perpetrators and victims of extreme violence (Winter et al., 2016), and in elucidating the different pathways to radicalization of Jihadi terrorists in India (Sarangi et al., 2013). Also of possible relevance to the experiences of individuals prior to radicalization is that grid indices of difficulty in construing oneself and others have been related to measures of hopelessness (Winter D. et al., 2007) and alienation (Winter et al., 2009).

The usual procedure for individual administration of the repertory grid is to ask the person to provide a list of elements of his or her world, normally aspects of the self and significant others; to give constructs by comparing and contrasting groups of these elements; and then to rate all of the elements on each of the constructs thus elicited. A variation on this procedure which has been tailored to the exploration of radicalization and deradicalization (Winter, 2010) uses a set of elements consisting of members of the radical group, including the self after radicalization; another set consisting of people who are not members of this group, including the self before radicalization; and the ideal self. Constructs are then elicited separately from the “radical” and the “non-radical” elements. With the aid of computer software, it is possible to derive from such a grid measures of: (1) increments in the structure of the construct system and predictability of the world when an individual adopts a radicalized rather than a non-radicalized perspective; (2) reduction in dilemmas and conflicts (Feixas and Saúl, 2004; Bell, 2014) in the radicalized as compared to the non-radicalized self; (3) increase in self-esteem when the radicalized rather than the non-radicalized perspective is adopted; and (4) relative positivity of the individual’s construing of radicalized as compared to non-radicalized people. More specifically, these grid measures include: (1) the relative intercorrelations between each set of constructs (“radical” and “non-radical”) and their relative sums of squares (the extent to which they discriminate between the elements), together with the relative sums of squares (indicating salience or meaningfulness) of the radical and non-radical elements; (2) measures of logical inconsistency in the construing of the radicalized and non-radicalized self; (3) relative dissimilarity in the construing of the radicalized and non-radicalized selves and that of the ideal self; (4) relative dissimilarity in the construing of radicalized and non-radicalized people and that of the ideal self.

Another assessment method that Kelly (1955) devised as an application of his credulous approach is the self-characterization, in which the person is asked to write an autobiographical description as if it were written by someone who knows him or her well and is sympathetic. A variation on this procedure which might be useful in the study of radicalization would be the writing of descriptions of the self before and after radicalization. Analysis of a self-characterization will consider both its organization and its content, but a particular focus is likely to be on the constructs used within it. PCT concepts (or, as Kelly termed them, professional constructs) are then applied to produce a formulation (Winter and Procter, 2013) based on the self-characterization and any other relevant material. As described by Feixas and Villegas (1991), longer pieces of text may be converted into, and analyzed as, repertory grids, a method that has been used to analyze books written by a serial killer (Winter D. A. et al., 2007) and the Commandant of Auschwitz concentration camp (Reed et al., 2014).

There are several other personal construct, or more broadly constructivist, assessment methods (Neimeyer, 1993; Caputi et al., 2012; Bell, 2016). Some of these focus on change in construing or resistance to such change, and therefore may be particularly relevant to investigating the reconstruing involved in radicalization and deradicalization. For example, Experience Cycle Methodology (Oades and Viney, 2012) is a structured interview that considers the stages that the individual has gone through in testing out and, if necessary, revising their constructions. The ABC model (Tschudi and Winter, 2011), by considering the positive and negative implications of each pole of a construct, may elucidate the individual’s choices and the reasons why it may be difficult for him or her to change.

We shall now illustrate how some of these approaches have been used in investigating the personal construct model of radicalization.

## Investigations of the Personal Construct Model of Radicalization

### A Study of Salafist Muslims

This study investigated young people in Tunisia who had identified with Salafism, a branch of Sunni Islam that calls for a return to the practices of the first Muslims (Muhanna-Matar and Winter, 2017; Winter and Muhanna-Matar, 2019). While some Salafists are committed to a path of Jihad, others reject the use of violence.

Five male and one female participants, with a mean age of 26.3 years, were recruited from young people who took part in a larger study using a narrative interview on the basis that they were willing to complete a repertory grid in addition to the interview. Two of them had become deradicalized. The grid that they completed followed the procedure described above developed by Winter (2010).

As described by Winter and Muhanna-Matar (2019), the narrative interviews clearly indicated the cycles of construing and reconstruing (involving Kelly’s Experience, Creativity, and Circumspection-Preemption-Control Cycles as well as oscillation between constriction and dilation) in which the participants had engaged on their paths to radicalization and, in two cases, deradicalization. While the interviews thus elucidated construing processes, the repertory grid was able to indicate features of the structure and content of the participants’ construing consistent with the personal construct model. Specifically, it was found that participants’ self-constructions as members of the group with which they currently identified were significantly less conflictual and more positive than their self-constructions as non-members of this group. They also construed members of the group with which they identified significantly more positively than non-members. The grid therefore complemented the interviews by providing further evidence that identification with a particular group allowed each of these young people to develop a more certain and favorable construction of the self.

These findings can be illustrated by considering two participants, one who remained radicalized and another who had become deradicalized. The grid of the former participant, Sami, who since becoming radicalized has “never felt happy like how I feel now… I have found a balanced style of life” (Muhanna-Matar and Winter, 2017, p. 397), indicated that Salafists, including himself as a Salafist, were much more salient, favorably construed, and associated with less conflict (and therefore more certainty) in his construing than non-Salafists, including himself before radicalization. This example can be contrasted with the grid of Saleem, who became deradicalized after following the path of Jihad to Syria and experiencing the major invalidation of finding himself fighting another Jihadist group, including his friend. As he said, “I went to Syria to join Jihadists who fight against the Bashar regime, not to fight against each other” (Muhanna-Matar and Winter, 2017, p. 405). He now considered that “I have become a different person” (p. 407). In his grid, non-Salafists were more salient, more favorably construed, and associated with less conflict than Salafists. There were also more dilemmas in his construing of his Salafist than non-Salafist self. For example, he viewed the former as “socially isolated,” “quick tempered,” and “pessimistic” (his own words), and although his preference was to be “sociable,” “sober minded,” and “optimistic,” his dilemma was that he associated these preferred characteristics with such negative implications as being “careless” and “fearful.”

### A Study of a Mass Murderer, Anders Breivik

Before setting out on a “mission” in which he killed 77 people, mostly at a Labor party youth camp, the Norwegian terrorist Anders Breivik circulated on the internet a 1,516-page compendium entitled “2083: A European Declaration of Independence” (Winter and Tschudi, 2015). A 66-page section of his compendium, described as an interview with a “Justiciar Knight Commander of the PCCTS Knights Templar,” is clearly an interview of Breivik by himself, and as such (particularly 38 pages devoted to his “personal life and convictions”) is similar to a self-characterization.

Breivik’s interview with himself was analyzed as if it were a self-characterization by extracting constructs from it and viewing it in terms of the professional constructs of PCT. We shall now map material from the “interview” and other information about Breivik onto the PCT model of radicalization.

1. *The radicalized individual has a history of invalidation of his/her construing, particularly in regard to core aspects of self-construing*.
  From childhood to adolescence, and in his later ventures into politics and business, Breivik seemed to have suffered a long series of invalidations (Winter and Tschudi, 2015), although he minimized these. For example, despite childhood events including his parents’ divorce, a battle for his custody, his father’s second divorce, and loss of contact with his father, he said that he did not have “any negative experiences in” his “childhood in any way” (Berwick, 2011, p. 1387), reserving criticism for the sociopolitical systems he saw as influencing his family. His experiences when he attempted to dedicate his life to politics included invalidation of his view that it would be possible to change the system democratically. His business ventures were no more successful in that he went bankrupt, but he was able to reconstrue this apparent invalidation as a cost-efficient way of raising money for new ventures.
2. This sometimes involves one or more episodes that lead to massive invalidation, and act as “transformative triggers.”
  A particularly significant episode of invalidation in adolescence seems to have been the rejection of “the Norwegian way” by his best friend, a Muslim, who began to assault and harass ethnic Norwegians. However, he regards the episode that “tipped the scales” for him as the Norwegian government’s support of the bombing of Serbia when “all” the Serbs “wanted” was “to drive Islam out” (Berwick, 2011, p. 1380).
3. *The individual with a very undifferentiated (and thus inflexible) construct system may be particularly vulnerable to such invalidation and consequent structural collapse*.
  Undifferentiated construing may be indicated by psychiatric reports that Breivik as a child was cautious, controlled, and lacked spontaneity, imagination, and empathy (Seierstad, 2015).
4. *His/her radical beliefs, usually drawing upon available social constructions, allow the development of a “turning point” in his or her sense of identity with a more structured and certain view of the world*.
  Breivik’s radical beliefs led to re-establishment of himself “on an existential level” as more moral and responsible than the “self centred fuck” he used to be (Berwick, 2011, p. 1406). As he said in relation to his ethnic Norwegian “brothers and sisters,” “my love for them exceeds my own self serving interests. That’s not the kind of person I used to be, but it’s the kind of person that I have become” (Berwick, 2011, p. 1403). In contrast to his previous existence of “pretty shallow ambitions… in a society in complete moral decay where you are detached from your extended family, your community, the Church and with little national and cultural identity and pride etc.,” (Berwick, 2011, p. 1402), he considered that “I have never been happier than I am now” (p. 1403).
  As described by Winter and Tschudi (2015), the choices that Breivik made were elucidated by applying an ABC model analysis to his description of the relative advantages and disadvantages of the alternative options of “completely focusing on tasks as part of the European resistance movement” or “creating a large family.” While he viewed the latter option as involving “living an easy life” and “avoiding suffering,” the former enabled him to see himself as a “good”, “selfless” man rather than a “guilty” “coward”, a “miserable creature” and “apathetic hypocrite.”
5. *The development of an extreme negative construction of another group, which may be perceived as responsible for the individual’s invalidations, allows further definition of the self by contrast with this group*.
  The vilification of cultural Marxists allowed him to see himself as “a destroyer of multiculturalism, … a destroyer of evil and a bringer of light” (Berwick, 2011, p. 1435). The threats that he had experienced were attributed to the forces of multiculturalism, and, as he wrote, “Fighting for your people’s survival, when threatened, is the most logical thing to do.”
6. *The individual’s radical constructions are validated by contact with others who share similar views, often coupled with constriction of their previous social world to avoid further invalidation*.
  Breivik essentially constricted his world by retiring to his bedroom for several years, initially to play war games and then to research on the internet the material that he used in writing his compendium. He described this as “a process I used in order to isolate myself from most of my network…. to completely ‘detach myself from ‘the game’, my ‘former shallow consumerist lifestyle’” (Berwick, 2011, p. 1381). During this period, he tried to make email contact with those who shared similar views.
7. *The likelihood of acting upon radical beliefs, including violent actions, is greater in those individuals in whom beliefs in such actions provide the greatest increment in the structure of his/her view of the self*.
  It seems likely that, for Breivik, violent action enabled him to achieve the ultimate further structuring of his identity as “The Perfect Knight I have always strived to be” (Berwick, 2011, p. 1435), as well as ensuring that his views reached a much wider audience, and potential sources of validation, than those to whom he sent his “European Declaration of Independence.”
8. *Reconstruing of violence as acceptable may be necessary if the person is to engage in such acts without guilt (and indeed to experience guilt for not engaging in them).*
  Breivik construed himself as someone who “wouldn’t be willing to hurt a fly and… never used violence against others” (Berwick, 2011, p. 1395). Nevertheless, he seemed able subsequently to avoid guilt following his murderous actions by maintaining that “I acted out of goodness, not evil… under normal conditions I am a very nice person.” For him, “There are situations in which cruelty is necessary, and refusing to apply necessary cruelty is a betrayal of the people whom you wish to protect” (Berwick, 2011, p. 846). Therefore, according to his beliefs, what would have been likely to cause him to feel guilt would have been failing to commit the murders.
9. *His/her radical view of the world may be shored up by “hostility,” in* *Kelly (1955* *) sense of extorting evidence for the individual’s constructions*.
  Breivik’s hostility (in Kelly’s sense) was evident in such statements as that if constructions of Muslims as violent were not validated, this could “be achieved by provoking and inciting them to choose the path of Jihad prematurely,” by “pin prick attacks” on “their most prized ‘possessions’, their women” (Berwick, 2011, p. 930).
10. *Similar processes may operate in members of the “other” group, creating a vicious cycle of extreme construing based on mutual validation of extreme negative views of the other*.
  Breivik made it clear that his aim was to create a “spiral” of radicalization which “will polarize societies.”

## Advantages and Limitations of the Personal Construct Model

The studies of Salafist Muslims and of Anders Breivik, while using different methodologies, have both provided findings consistent with the personal construct model of radicalization. This model can be considered to have numerous benefits. Firstly, similar to personal construct approaches in other fields, such as psychotherapy (Neimeyer, 1988), the model is sufficiently permeable and holistic to integrate numerous existing models. It can encompass both individual and, as indicated by Procter (2016), societal processes of construing, and suggests predisposing factors to radicalization at individual and societal levels. Importantly, it explains the choices and actions of the radicalized individual in terms of the same processes of construing as operate in any other personal choice, and therefore does not pathologize the radicalized person. Similarly, its “credulous attitude” is concerned with understanding, rather than condemning and “waging war upon,” the radicalized person’s view of the world. The model explains both radicalization and deradicalization in terms of similar processes, and, by drawing upon methods used in personal and relational construct psychotherapy (Winter and Viney, 2005; Procter and Winter, 2019), can offer a range of interventions for facilitating reconstruing which could be used in individual tailoring of deradicalization programs. Detailing such approaches is beyond the scope of the present paper, but they could include interventions designed to develop an alternative structured view of the world and/or focused on the resolution of dilemmas associated with becoming deradicalized (Feixas, 2016). Perhaps most significantly, the personal construct model has an associated methodology which allows empirical exploration of the construing of radicalized people and monitoring of changes during deradicalization. This methodology combines the advantages of both qualitative and quantitative approaches, and can be applied not only in an individual interview format but also to the construing of groups and to textual material.

The model’s primary limitation is that the evidence on which it is based is limited. Further research is therefore necessary, and current investigations include studies of the relationships between measures derived from the repertory grid method developed for the study of radicalization and measures of degree of identification and “fusion” with the groups to which individuals are affiliated. The model will also be tested by deductive thematic analysis of material provided by people who have been radicalized.

At present, however, the model can at least be regarded as offering a heuristic that provides a way of understanding and empirically investigating the choices involved in radicalization and deradicalization which, in the words of Taylor and Horgan (2006), assumes that radicalized individuals and “terrorists are ordinary people (to the extent that they are not distinguishable from other “ordinary” people) who make choices in the contexts in which they find themselves” and “that choices made by individuals are meaningful for the person making those choices” (p. 588).

## Author Contributions

Both authors planned and discussed the objectives and structure of the paper, and shared responsibility on the final version of the manuscript. DW wrote an initial draft and the section on his own research. GF produced Figure 1 and incorporated in it the feedback provided by DW.

### Conflict of Interest Statement

The authors declare that the research was conducted in the absence of any commercial or financial relationships that could be construed as a potential conflict of interest.
